# The Effect of Carnosine Supplementation on Musculoskeletal Health in Adults with Prediabetes and Type 2 Diabetes: A Secondary Analysis of a Randomized Controlled Trial

**DOI:** 10.3390/nu16244328

**Published:** 2024-12-15

**Authors:** Saeede Saadati, Paul Jansons, David Scott, Maximilian de Courten, Aya Mousa, Jack Feehan, Jakub Mesinovic, Barbora de Courten

**Affiliations:** 1 Monash Centre for Health Research and Implementation (MCHRI), Faculty of Medicine, Nursing and Health Sciences, Monash University, Clayton, VIC 3168, Australia; saeede.saadati@monash.edu (S.S.); aya.mousa@monash.edu (A.M.); 2 Department of Medicine, School of Clinical Sciences, Faculty of Medicine, Nursing and Health Sciences, Monash University, Clayton, VIC 3168, Australia; paul.jansons@deakin.edu.au (P.J.); d.scott@deakin.edu.au (D.S.); jakub.mesinovic@deakin.edu.au (J.M.); 3 Institute for Physical Activity and Nutrition (IPAN), School of Exercise and Nutrition Sciences, Deakin University, Geelong, VIC 3220, Australia; 4 Australian Health Policy Collaboration, Institute for Health and Sport (IHES), Victoria University, Melbourne, VIC 8001, Australia; maximilian.decourten@vu.edu.au; 5 School of Health and Biomedical Sciences, RMIT University, Bundoora, VIC 3083, Australia; jack.feehan@rmit.edu.au

**Keywords:** musculoskeletal health, carnosine, prediabetes, type 2 diabetes, randomized trial, RCT, insulin resistance, body composition, physical function, bone health

## Abstract

Background/Objectives: Type 2 diabetes (T2D) is associated with an increased risk of adverse musculoskeletal outcomes likely due to heightened chronic inflammation, oxidative stress, and advanced glycation end-products (AGE). Carnosine has been shown to have anti-inflammatory, anti-oxidative, and anti-AGE properties. However, no clinical trials have examined the impact of carnosine on musculoskeletal health in adults with prediabetes or T2D. Methods: In a randomized, double-blind clinical trial, 49 participants with prediabetes or T2D and without existing musculoskeletal conditions were assigned to receive either 2 g/day carnosine or matching placebo for 14 weeks. Whole-body dual-energy X-ray absorptiometry (DXA) was used to assess body composition, and peripheral quantitative computed tomography (pQCT) was used to assess bone health at the distal and proximal tibia. Results: Forty-three participants completed this study. Carnosine supplementation had no effect on change in hand grip strength (HGS) or upper-limb relative strength (HGS/lean mass) versus placebo. Change in appendicular lean mass, percentage of body fat, visceral fat area, proximal tibial cortical volumetric bone mineral density (vBMD), distal tibial trabecular vBMD, and stress-strain index did not differ with carnosine compared to placebo. Fourteen weeks of carnosine supplementation did not improve muscle strength, body composition, or bone health in adults with prediabetes or T2D. Conclusions: Carnosine supplementation may not be an effective approach for improving musculoskeletal health in adults with prediabetes and T2D without musculoskeletal conditions. However, appropriately powered trials with longer duration are warranted to confirm our findings. The trial was registered at clinicaltrials.gov (NCT02917928).

## 1. Introduction

Type 2 diabetes (T2D) is a serious public health concern with global prevalence projected to increase from 5.9% in 2021 to 9.5% in 2050, where it will affect more than 1.27 billion people [1]. In addition to micro- and macrovascular complications, individuals with T2D are at increased risk for adverse musculoskeletal outcomes such as falls, fractures, and disability [2]. Major contributors to these outcomes include T2D-related declines in muscle strength and muscle mass and impairments in bone material properties and structure [3,4].

Despite having higher body mass and bone mineral density (BMD), individuals with T2D still have higher fracture rates [5]. Several purported mechanisms explain the relationship between poor musculoskeletal health and T2D. Given the anabolic action of insulin on skeletal muscle, insulin resistance, a hallmark of T2D pathogenesis, may result in reduced protein synthesis and elevated protein degradation leading to the loss of muscle mass, strength, and function [6,7]. Similarly, chronic hyperglycemia increases advanced glycation end-products (AGEs), inflammatory cytokines, and oxidative stress, which accelerate declines in muscle mass, strength, and function [8,9]. AGEs have been shown to accumulate within the bone matrix, leading to impairments in bone material properties and structure that ultimately impair bone turnover and strength, independent of BMD [10,11,12,13,14]. The presence of vascular complications, including diabetic kidney disease [15,16], peripheral diabetic neuropathy [17,18], diabetic retinopathy [19,20], and peripheral vascular disease [21,22], also contributes to increased fall and fracture risk in T2D. Therefore, interventions targeting both metabolic and musculoskeletal health might be beneficial for this population. Utilizing technologies other than dual-energy X-ray absorptiometry (DXA; which only measures areal BMD), such as peripheral quantitative computed tomography (pQCT), can help identify compromised bone structural characteristics in individuals with T2D [23,24].

Adherence to guidelines for lifestyle modifications (healthy diet and physical activity) is effective for managing T2D and its associated musculoskeletal health complications [8,25,26]. However, a US study showed that adults with diabetes are less likely to participate in physical activity at recommended levels compared to those without diabetes (40.2–42.9 vs. 48.0–51.5%) [27]. Due to socioeconomic and environmental factors, adhering to lifestyle changes is challenging for many people, in particular, for patients with chronic diseases such as T2D. This highlights the need for adjunct therapies that are safe, effective, and sustainable over the long term [28].

Carnosine, also known as β-alanyl-L-histidine, belongs to the family of histidine-containing dipeptides (HCDs) and is an endogenous dipeptide with well-demonstrated pharmacological activities, including anti-inflammatory, antioxidant, and anti-AGE properties [29]. Carnosine and its methylated derivatives are present with the highest concentration in skeletal and cardiac muscles [29]. Carnosine might maintain or improve musculoskeletal health by reducing protein catabolism, promoting the proliferation and differentiation of osteoblasts, protecting chondrocytes, and inhibiting osteoclasts [30,31,32,33]. Carnosine helps preserve muscle mass and function, particularly under conditions of stress or injury, by reducing protein breakdown. It also supports bone health by enhancing osteoblast activity, which contributes to bone formation and mineralization, and by inhibiting osteoclast-mediated bone resorption, ensuring a healthy balance in bone remodeling. Additionally, carnosine’s protective effects on chondrocytes may help prevent cartilage degeneration, a key factor in joint health [30,31,32,33]. It may also indirectly protect against musculoskeletal decline by modulating cardiometabolic health, such as via improving glycemic control [34] and reducing chronic low-grade inflammation, oxidative stress, lipid peroxidation [35], and AGEs [36]. Despite biologically plausible benefits, to date, no randomized placebo-controlled trials have explored the effects of carnosine on musculoskeletal health.

To address this knowledge gap, we conducted a secondary analysis of a previous RCT to explore the effects of 14 weeks of carnosine supplementation on body composition, muscle strength, and bone structure in adults with prediabetes and T2D.

## 2. Materials and Methods

### 2.1. Study Design and Participant Recruitment

This is a secondary analysis of a 14-week parallel-group, single-site (Monash Health Translational Research Facility, Melbourne, Australia), randomized, double-blind, placebo-controlled trial [34,37,38]. Participants were recruited via community advertising at Monash Medical Centre and Monash University in Melbourne, Australia, as well as the Australian National Diabetes Service Scheme. Eligible participants were 18–70 years old and diagnosed based on the World Health Organization criteria with a 75 g oral glucose tolerance test (OGTT) as having prediabetes (fasting blood glucose of 6.1–6.9 mmol/L, or 2 h blood glucose of 7.8–11.1 mmol/L following OGTT) or T2D (fasting blood glucose ≥ 7.0 mmol/L and 2 h blood glucose ≥ 11.1 mmol/L), which was diet-controlled, metformin-treated, or untreated, and with a hemoglobin A1C (HbA1C) concentration < 8%. Exclusion criteria included a body mass index (BMI) > 40 kg/m^2^; smoking; high alcohol use (>4 standard drinks/week for men and >2 standard drinks/week for women); weight change of >5 kg in the last 6 months; being pregnant or breastfeeding; using dietary supplements or medications known to affect cardiometabolic measures; renal failure (estimated glomerular filtration rate [GFR] of <30 mL/min); cardiovascular, endocrine gastrointestinal, hematological, central nervous system, or respiratory diseases; active cancer; psychiatric disorders; acute inflammation or infection within the previous 5 years; or any history of blood transfusion within the last three months. This study included one screening visit and two clinic visits, one at baseline and the other repeated after the intervention. As this was a secondary analysis, there was no a priori power calculation, which makes it likely that it was underpowered.

### 2.2. Ethics

The trial was prospectively registered (ClinicalTrials.gov identifier: NCT02917928, 28/09/16) and approved by the Monash University Human Research Ethics Committee (ID number: 7787) and Monash Health (Ref. No. 16061A) in Melbourne, Australia. The trial was performed according to the principles of the Declaration of Helsinki [39] and the Standardised Protocol Interventions: Recommendations for Interventional Trials (SPIRIT) 2013 Statement [40] and is reported according to CONSORT guidelines [41].

### 2.3. Screening Visit

After obtaining written informed consent, a registered general practitioner completed the baseline medical history assessment and all anthropometric outcome measures. A urine pregnancy test was also performed to exclude pregnancy. Participants then completed a 2 h 75 g OGTT to confirm the presence of prediabetes or T2D based on the World Health Organization criteria outlined above [42].

### 2.4. Randomization, Intervention, Adherence, and Adverse Events

Following the initial screening visit and baseline testing, eligible participants were randomized to either the intervention group, who were instructed to orally consume carnosine (two capsules of 500 mg, twice daily for 14 weeks) (CarnoPure, Flamma S.p.A, Bergamo, Italy), or the placebo group, who were given an equivalent number of indistinguishable placebo capsules for 14 weeks. Participants, researchers, outcome assessors, and this study statistician were all blinded to group allocation. Random sequence assignment was performed in blocks of four stratified by gender and metformin intake status (yes/no) using a computerized random sequence-generation program. The research statistician created the randomization codes, which were then sent to a Clinical Trial Pharmacy for allocation and dispensing. To ensure double-blinding, treatments were dispensed in identical, flavorless, non-transparent capsules stored within opaque containers. Adherence to the intervention was achieved by checking returned carnosine containers and counting the number of remaining capsules.

All participants were supported to consume the desired carnosine dosage via monthly telephone calls by study investigators over the 14-week study period. Carnosine supplements used in this study had a purity greater than 99.5% and were entirely synthetic, odorless, and crystalline. The selection of a 2 g/day dosage was based on previous preliminary human trials on insulin secretion and sensitivity [43]. All participants were asked to maintain their usual diet and physical activity while participating in this study.

Adverse events associated with carnosine supplementation (rash, dry mouth, and/or feelings of tiredness) were assessed during the monthly phone calls and followed up as needed. Participants were also asked to contact the trial physician if any adverse events occurred; these were documented and reviewed by the chief investigator.

### 2.5. Anthropometry

Body weight (kg) and height (cm) were measured using a digital scale (Tanita BWB-600, Tanita, Tokyo, Japan) and a wall-mounted stadiometer (Seca 206, Seca, Hamburg, Germany), respectively. BMI was calculated by dividing weight (kg) by the square of height (m^2^).

### 2.6. Musculoskeletal Assessments

Whole-body DXA (Hologic Discovery A, Hologic, Marlborough, MA, USA) was used to estimate body composition parameters, including fat and lean mass, body fat percentage (PFAT), and visceral fat area (VFAT area; cm^2^). Total lean mass in both the upper and lower limbs was summed to calculate appendicular lean mass (ALM), which was then corrected for height by dividing it by height (m^2^). Upper-limb relative muscle strength was calculated using the following formulas: the average hand grip strength (HGS) of the dominant arm (kg) divided by the lean mass (determined through DXA) in the corresponding limb (kg) and the average HGS of the dominant arm (kg) divided by body weight [44]. The DXA scanner was calibrated with the manufacturer’s spine phantom on a daily basis. The short-term intra-individual coefficients of variation (CV) of fat mass and ALM were 2.67% and 1.60%, respectively. All DXA scans were conducted by two researchers (J.M. and P.J.).

A single 2.5 mm transverse pQCT scan (Stratec XCT3000, Stratec Medizintechnik GmbH, Pforzheim, Germany) was performed at a speed of 20 mm/s and a voxel size of 0.8 mm. Scans were obtained at 4% (distal) and 66% (proximal) of the tibial length (relative to the distal end) of the non-dominant leg. The tibial length was measured by the distance between the prominence of the medial malleolus and the tibial plateau. A planar scout view of the distal tibia was employed to determine scan sites, and reference lines were placed parallel to the distal joint surface of the tibia [45].

All scans were analyzed using the manufacturer’s algorithms and software (version 6.2). The trabecular bone at the distal tibia was identified using the default setting of the manufacturer, wherein the outer 55% of the bone area is concentrically separated, designating the inner 45% as trabecular bone. While the trabecular bone compartment is larger than 45% in this region, the limitations in pQCT resolution make it challenging to easily distinguish the boundary between cortical and trabecular bone in this area. Therefore, characterizing the inner 45% of this region as trabecular bone is a conservative approach, providing a margin of safety to ensure that cortical bone is not mistakenly included in the trabecular region of interest [46]. Cortical bone was identified at the proximal radius and tibia using the default threshold of 710 mg/cm^3^ [45]. After applying these thresholds, various indicators related to bone density and structure were determined: total and trabecular volumetric bone mineral density (vBMD; mg/cm^3^) at the distal tibia and total cross-sectional area (CSA; mm^2^), cortical area (mm^2^), cortical vBMD (mg/cm^3^), and polar stress-strain index (SSI; mm^3^) [47] at the proximal tibia. Calibration of the device was performed daily using the manufacturer’s phantom, and the short-term intra-individual CVs for total vBMD, trabecular vBMD, and cortical vBMD were 1.2%, 2.4%, and 0.4%, respectively. Additionally, SSI, total CSA, and total cortical area had a CV of 3.2%, 0.7%, and 1.4%, respectively. All pQCT scans were examined by two researchers (J.M. and P.J.).

### 2.7. Physical Function

A Jamar Plus digital hydraulic hand grip dynamometer (Patterson Medical, Bolingbrook, IL, USA) was used to measure the HGS of the dominant hand [48]. To measure HGS, participants were asked to sit with their arms horizontally extended at their shoulder height and grip the dynamometer with maximum force for three seconds. This test was repeated three times in the dominant hand with a 60 s break between each trial. The average hand grip strength (kilogram) was determined by calculating the mean value from the second and third trials.

### 2.8. Clinical and Biochemical Assessments

After fasting for at least 10 h overnight, blood samples were collected using aseptic methods to evaluate glucose, insulin, full blood count, HbA1c, and lipid profile. All samples were analyzed by the National Association of Testing Authorities-accredited Monash Health pathology service. Systolic and diastolic blood pressures (SBP and DBP) were assessed with an automated oscillometric system (Omron BBP-742, Kyoto, Japan), following a 20 min seated rest, and the results were recorded as the average of three readings. CVs were 1.9–2.1% for plasma glucose, 4.6–5.0% for insulin, 0.8–1.1% for hemoglobin A1c (HbA1c), 1.5–2.2% for triglyceride (TG), 1.9–2.5% for total cholesterol (TC), and 1.9–2.8% for high-density lipoprotein cholesterol (HDL-C). Low-density lipoprotein (LDL) levels were calculated using the following formula: LDL-C = TC − HDL-C − (TG/2.2). The homeostatic model assessment for insulin resistance (HOMA-IR) was calculated using the following formula: fasting insulin concentration x fasting glucose concentration/22.5.

### 2.9. International Physical Activity Questionnaire (IPAQ)

Physical activity levels were assessed using the short form of the International Physical Activity Questionnaire (IPAQ). This version includes seven questions addressing the time spent on vigorous activities (e.g., aerobics), moderate activities (e.g., carrying small loads), walking, and sedentary behavior during the previous 7 days [49].

### 2.10. Record of Habitual Diet

Dietary intake, including food groups, macronutrients, micronutrients, and energy, was evaluated using 3-day food records collected over three consecutive days (two weekdays and one weekend day) both before and after the intervention. The records were analyzed using Foodworks.online.V.2.0 Professional Dietary Software (Brisbane, Australia, Xyris Pty Ltd.), along with Australian food composition data (NUTTAB 2010). 

### 2.11. Statistical Analysis

Analyses were conducted per-protocol using SPSS Statistics version 24 (IBM, Armonk, NY, USA). Normality assessments were performed for continuous data using boxplots and Shapiro–Wilk tests. If normality was violated, continuous variables were transformed by natural logarithm. Descriptive statistics are presented as means ± standard deviation (SD), frequencies (percentages), or as median (interquartile range [IQR]) for skewed distributions. Changes at 14 weeks for all outcomes and group-by-time interactions were analyzed using linear mixed models (LMMs) with an unstructured covariance matrix. LMMs were chosen because they are well-suited for repeated measures designs, as they account for the correlation between measurements taken from the same individual and allow for the inclusion of all available data, even in the presence of some missing values. Models included time (2 levels), group (2 levels), and a group-by-time interaction term as fixed effects, with unique participant identifiers as a random intercept. At the distal and proximal tibia, eight participants from both groups had poor-quality pQCT scans (grade = 4 [worst]) due to movement artifacts. Three participants in the carnosine group had poor-quality DXA scans. All participants with poor-quality scans had scan data imputed via linear mixed models to maximize statistical power. *p*-values < 0.05 were considered statistically significant.

## 3. Results

**Figure 1:**
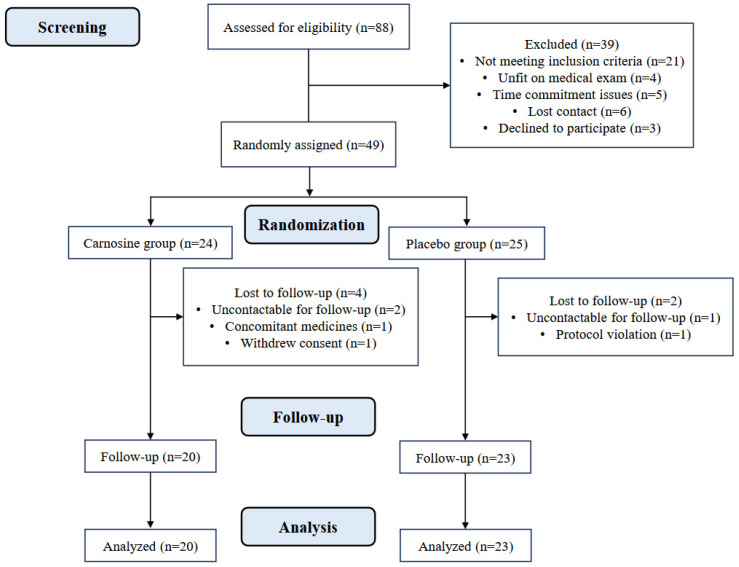
Study design and flow of participants.

A total of 88 participants were screened for eligibility, 21 of whom did not meet the inclusion criteria. Of the remaining 67 participants who attended a medical review, 18 participants were excluded prior to randomization due to being unfit for medical exams, time commitment issues, losing contact, or declining to participate. A total of 49 participants were randomly assigned to either the carnosine (*n* = 24) or placebo group (n = 25). At follow-up, six participants dropped out due to various reasons, including loss of interest, lost contact for follow-up, and protocol violation. Forty-three participants (carnosine n = 20; placebo n = 23) completed the trial (Figure 1). All 43 participants (carnosine n = 20; placebo n = 23) returned empty containers; therefore, we considered all participants to be 100% adherent to the intervention. Table 1 provides an overview of the baseline demographic, anthropometric, and biochemical characteristics of both groups. The cohort consisted of 30 males and 13 females who were aged 53 years (IQR: 44.2–59.4) with a mean BMI of 29.3 ± 4.3 kg/m^2^ and a HbA1C of 6.6 ± 0.7%. There were no supplement-related adverse events reported in this study.

Mean baseline values and between-group differences in body composition and muscle strength outcomes after 14 weeks of carnosine supplementation are presented in Table 2. After the 14-week intervention, there were no significant between-group differences in change in average HGS or relative muscle strength. There were also no differences in any body composition measures, including changes in BMI, PFAT, ALM, ALM/height^2^, and VFAT area.

Table 3 presents baseline values and between-group differences in bone densitometric and structural outcomes after 14 weeks of carnosine supplementation. Changes in total and trabecular vBMD at the distal tibia did not differ between groups. Similarly, no between-group differences in cortical vBMD or SSI in the proximal tibia were observed at the proximal site. There were also no differences in total cross-sectional area or cortical area at the proximal tibia.

## 4. Discussion

This was the first randomized controlled trial (RCT) to explore the effects of oral carnosine on body composition and musculoskeletal health using DXA and pQCT in adults with prediabetes and T2D. We demonstrated that 14 weeks of carnosine supplementation (2 g daily) had no significant effects on muscle strength, body composition, or bone health in individuals with prediabetes and T2D compared with placebo. There were no supplement-related adverse events during the 14-week intervention period.

While no preclinical or clinical studies have directly examined the effect of carnosine on muscle strength in individuals with prediabetes and T2D, existing evidence suggests that carnosine may have potentially beneficial effects on reducing fatigue and improving physical function among athletes [50] and older adults [51,52]. This beneficial effect likely occurs via the enhanced pH buffering effects of carnosine [53,54]. During exercise, lactic acid production increases, leading to the dissociation of lactate and H^+^, thereby reducing pH levels [55]. Carnosine mitigates acidosis by binding the imidazole group of histidine to a proton, preventing a drop in pH [53,56]. Carnosine could be used as a quick buffer during exercise as its dissociation constant (pKa) is closer to physiological pH [57]. The improvement in physical function associated with increased carnosine levels may also be related to enhanced excitation–contraction coupling by increasing local calcium recruitment [58]. Additionally, β-alanine supplementation, which increases muscle carnosine content, has been shown to improve physical function outcomes in older adults [51,52]. Carnosine and acetyl-carnosine levels, which are red blood cell (RBC)-enriched and a plasma compound, respectively, decline in older adults [59]. This could explain the improvement observed in older adults following carnosine supplementation. In the current study, the lack of effects for carnosine may be due to the younger cohort (median age: 53 years) and/or the relatively normal levels of muscle strength in most participants at baseline. This means that improvements in this population, if any, would be small, thus requiring a large sample size to detect. The small number of participants included in this study is likely underpowered to detect this potentially small effect. Further, it is possible that the 14-week duration of the trial was insufficient to observe beneficial effects on these outcomes, which likely require a longer follow-up duration to demonstrate measurable improvement. It is also possible that carnosine/HCDs do not improve muscle or physical function, as shown in other studies where carnosine did not improve muscle function in an experimental model of multiple sclerosis [60] and β-alanine supplementation did not change physical function during a 6-week training period in healthy men [61]. Confirmation of any potential benefits of carnosine/HCDs for physical function awaits further study.

The present study did not find significant changes in subtotal PFAT, VFAT area, ALM, and ALM/height^2^ following carnosine supplementation. The existing literature presents mixed findings regarding the effects of carnosine on body composition. In a 12-week pilot study, carnosine supplementation (2 g/day) did not improve PFAT or BMI in individuals with overweight and obesity [43]. Similarly, a systematic review and meta-analysis of 20 RCTs (492 adults) showed that administration of β-alanine did not affect body composition parameters, including body mass, fat mass, fat-free mass, and PFAT [62]. In contrast, in a case-control study of patients with upper gastrointestinal cancer, low muscle carnosine concentrations were associated with muscle wasting [63]. This finding suggests that low carnosine concentrations could alter the pH buffering capacity of muscle and antioxidant defenses, thus potentially contributing to muscle atrophy [63]. In several experimental studies and some RCTs, carnosine/HCD supplementation suppressed fat accumulation [64], fat mass, fat-free mass, and non-esterified fatty acids (NEFA) [36,65] and increased lean body mass [66]. Carnosine may decrease fat mass by stimulating irisin, a myocyte that increases thermogenesis in adipocytes [67]. Future studies investigating the effects of carnosine on body composition should measure circulating myokines, including irisin, to further elucidate the effects of carnosine on the browning of white adipose tissue [67].

No improvements in bone density or structure were observed in this study. This is in contrast to an experimental model of T2D-induced osteoarthritis, where oral carnosine supplementation reversed the inflammatory response through the reactive oxygen species (ROS)/nuclear factor-kappaB (NF-κB) pathway, suggesting it has a chondroprotective effect [68]. Carnosine/HCDs support bone health by stimulating osteoblasts to synthesize bone proteins, encouraging the differentiation of pluripotent mesenchymal stem cells into osteoblasts and chondrocytes, and reducing bone resorption activity by inhibiting osteoclast function. [32]. Although we did not observe benefits for bone health post-supplementation in the present study, this is likely attributed to several factors, including our relatively small sample size (i.e., insufficient statistical power) and short intervention duration, which was likely inadequate to observe changes in bone parameters. It should also be noted that findings in many experimental studies cannot be translated into human studies, highlighting the need for further large-scale human studies with long-term follow-up. Another possible explanation is that we did not observe significant changes in other cardiometabolic health aspects, including vascular function, arterial stiffness, or inflammatory markers, following carnosine supplementation in the same cohort of participants with prediabetes/T2D. The only improvement observed was in glucose levels, not insulin levels, which was likely insufficient to impact musculoskeletal health [34,37,38].

Strengths of the present study include a rigorous methodology and a randomized double-blinded placebo-controlled design. Moreover, this was the first study to explore the effects of carnosine supplementation on musculoskeletal health assessed using DXA and pQCT in participants with prediabetes and T2D. However, this was a secondary analysis of an RCT where musculoskeletal health was not the primary outcome. This study therefore has a relatively small sample size and may be underpowered to detect differences in musculoskeletal outcomes. The small sample size also limited our ability to stratify results based on relevant baseline characteristics, such as musculoskeletal health, insulin resistance, and age. Moreover, the follow-up duration may be insufficient to observe changes, particularly for changes in bone parameters that have slow and incremental improvements. Additionally, missing data on physical activity and dietary protein intake limited our ability to include these potential confounding variables as covariates in the analysis, which may have influenced the observed outcomes. Another limitation is the assessment of supplementation compliance through self-reporting, as a few participants did not return supplement bottles. We assumed adherence to the supplementation protocol was high based on those who returned the containers and case notes reported by researchers throughout follow-up sessions. In addition, urinary carnosine levels and muscle carnosine contents were not measured in this study. Finally, we included participants with prediabetes and T2D, so the results of this study might not apply to other populations.

## 5. Conclusions

Carnosine supplementation (2 g daily for 14 weeks) had no beneficial effect on muscle strength, body composition, or bone health outcomes in participants with prediabetes and T2D. Future studies should be larger with longer follow-up durations when investigating the effects and mechanisms of carnosine on musculoskeletal health.

## Figures and Tables

**Table 1:** Participant demographics and baseline characteristics.

Characteristic	Carnosine Group (n = 20)	Placebo Group (n = 23)
Age, y	53.7 (48.5–59.4) ^a^	52.1 (42.3–59.3)
Men, n (%)	14 (70)	16 (70)
Caucasian	11 (55)	11 (47.8)
South and Central Asian	6 (30)	6 (26)
Southeast and Northeast Asian	3 (15)	5 (21.7)
Other ^b^	-	1 (4.3)
Prediabetes, n (%)	11 (55)	11 (47.8)
T2D, n (%)	9 (45)	12 (52.2)
Family history of diabetes ^c^, n (%)	5 (25)	4 (17.3)
Treated with metformin, n (%)	8 (35)	9 (30.4)
Total energy, kj	7935.8 ± 1303.6	8206.8 ± 1319.3
Physical activity ^d^, IPAQ-METS score ^e^	2400 (798–4878)	1332 (390–2736)
Weight, kg	88.4 ± 23.8	81.8 ± 14.5
Height, cm	172.3 ± 12.3	169 ± 10.3
BMI, kg/m^2^	29.8 ± 4.9	28.5 ± 3.7
Glucose AUC	45.8 ± 9.8	43.2 ± 7.5
Insulin AUC	257.5 ± 216.6	159.1 ± 113.3
HOMA-IR	4.1 ± 5.3	2.7 ± 1.7
HbA1c,%	6.5 ± 0.6	6.7 ± 0.8
TG, mmol/l	1.7 ± 0.9	1.7 ± 0.7
TC, mmol/l	5.4 ± 0.9	5.3 ± 1
LDL-C, mmol/L	3.4 ± 0.6	3.2 ± 0.6
HDL-C, mmol/L	1.1 ± 0.3	1.2 ± 0.3
SBP, mmHg	122.8 ± 14.8	122.7 ± 12.5
DBP, mmHg	80.6 ± 8.2	82.1 ± 7.1

^a^ Median; IQR in parentheses (applies to all values for non-normally distributed variables). Variables with non-normal distribution were log-transformed to the base 10 prior to analysis. ^b^ Refers to ethnicities including African, Middle Eastern, South American, and Polynesian ethnicities. ^c^ Includes only first-degree relatives with diabetes. ^d^ Calculated from self-reported questionnaires and food records. ^e^ IPAQ-METS, international physical activity questionnaire–multiples of the resting metabolic rate. Abbreviations: BMI: body mass index, glucose AUC: glucose area under the curve, HOMA-IR: homeostatic model assessment for insulin resistance, HbA1c: hemoglobin A1c, TG: triglyceride, TC: total cholesterol, HDL-C: high-density lipoprotein cholesterol, LDL-C: low-density lipoprotein cholesterol, SBP: systolic blood pressure, DBP: diastolic blood pressure, and T2D: type 2 diabetes.

**Table 2:** Mean baseline values and per-protocol analysis of between-group changes in body composition and physical function after 14 weeks in carnosine and placebo groups.

Outcome Variable	*Carnosine Group (n = 20)*			*Placebo Group (n = 23)*			Mean Difference ^a^	Group x Time (*p*-Value)
	Baseline	Follow-Up	Within-Group Change	Baseline	Follow-Up	Within-Group Change		
PFAT (%)	37.3 ± 8.1	37.1 ± 8	−0.1 ± 1.5	36.3 ± 7.4	35.9 ± 7.4	−0.4 ± 1.4	0.3 (−0.6, 1.1)	0.537
ALM (kg)	25.3 ± 7	25.1 ± 7	−0.2 ± 0.7	23.1 ± 6.4	23.1 ± 6.4	−0.04 ± 0.6	−0.1 (−0.6, 0.3)	0.455
ALM/height^2^ (kg/m^2^)	8.4 ± 1.3	8.3 ± 1.4	−0.07 ± 0.2	7.9 ± 1.2	7.9 ± 1.3	−0.01 ± 0.2	−0.06 (−0.2, 0.1)	0.440
VFAT Area (cm^2^)	144.1 ± 51.5	141.2 ± 47.8	−3 ± 16.7	149.9 ± 44.6	143 ± 41.4	−6.9 ± 14.5	4 (−5.8, 13.7)	0.413
Average HGS dominant (kg)	38.3 ± 12.2	38.4 ± 11.6	0.1 ± 4.1	37.2 ± 13.5	38 ± 12.7	0.8 ± 4.3	−0.7 (−3.3, 1.9)	0.594
Relative muscle strength (HGS/BW)	0.4 ± 0.1	0.4 ± 0.1	0.001 ± 0.06	0.4 ± 0.1	0.4 ± 0.1	0.007 ± 0.06	−0.005 (−0.04, 0.03)	0.769
Relative muscle strength (HGS/ARMLM)	0.6 ± 0.2	0.6 ± 0.2	0.004 ± 0.08	0.6 ± 0.2	0.7 ± 0.2	0.01 ± 0.08	−0.01 (−0.06, 0.04)	0.666

Data are mean ± SD or mean (95% confidence intervals). Bold indicates statistical significance (*p* < 0.05). ^a^ The mean difference refers to the difference in within-group changes between the carnosine group and the placebo group. Abbreviations: BMI: body mass index, PFAT: body fat percentage, ALM: appendicular lean mass, VFAT Area: visceral fat area, HGS: hand grip strength, BW: body weight, ARMLM: lean mass of the dominant arm.

**Table 3:** Mean baseline values and per-protocol analysis of between-group changes in bone-related outcomes after 14 weeks in carnosine and placebo groups.

Outcome Variable	*Carnosine Group (n = 20)*			*Placebo Group (n = 23)*			Mean Difference ^a^	Group x Time (*p*-Value)
	Baseline	Follow-Up	Within-Group Change	Baseline	Follow-Up	Within-Group Change		
***Distal tibia***								
Total vBMD (mg/cm^3^)	312.4 ± 48.8	312.9 ± 46.9	0.5 ± 19.2	312.4 ± 48	305.4 ± 46.1	−7 ± 18.9	7.4 (−4.4, 19.3)	0.212
Trabecular vBMD (mg/cm^3^)	253.3 ± 46.7	254 ± 42	0.7 ± 22.7	255.3 ± 45.9	250.5 ± 41.3	−4.8 ± 22.4	5.6 (−8.4, 19.6)	0.423
***Proximal tibia***								
Cortical vBMD (mg/cm^3^)	1102 ± 40.3	1100.6 ± 42.1	−1.5 ± 19.5	1106.8 ± 39.7	1104.9 ± 41.5	−1.9 ± 19.1	0.4 (−11.6, 12.4)	0.943
SSI	2503.8 ± 686.9	2423.2 ± 710.1	−80.5 ± 354.1	2311.8 ± 675.9	2249.5 ± 698.8	−62.4 ± 348.4	−18.1 (−236.8, 200.5)	0.867
Total cross-sectional area (mm^2^)	788.1 ± 238.5	844.4 ± 246.2	56.3 ± 170.4	738.8 ± 234.7	782.9 ± 242.3	44.1 ± 167.6	12.2 (−93, 117.4)	0.815
Total cortical area	327.4 ± 60.9	326.9 ± 62.2	−0.5 ± 12.6	300.7 ± 59.9	296.9 ± 61.3	−3.8 ± 12.4	3.2 (−4.5, 11)	0.404

Data are mean ± SD or mean (95% confidence intervals). Bold indicates statistical significance (*p* < 0.05). ^a^ The mean difference refers to the difference in within-group changes between the carnosine group and the placebo group. Abbreviations: vBMD: volumetric bone mineral density, SSI: stress-strain index.

## Data Availability

The datasets used and/or analyzed during the current study are available from the corresponding author upon reasonable request.
